# Exploring the Nexus of Healthcare Employees’ Professional Quality, Health Psychology and Service Value: A Qualitative Study

**DOI:** 10.3390/ijerph191912462

**Published:** 2022-09-30

**Authors:** Bailin Ge, Zhiqiang Ma, Mingxing Li, Xiaomeng Chi, Hira Salah ud din Khan, Ling Yang

**Affiliations:** 1 School of Management, Jiangsu University, Zhenjiang 212013, China; 2 Jingkou District Jiankang Road Community Health Service Center, Zhenjiang 212001, China

**Keywords:** healthcare employees, health psychology, professional quality, service value, grounded theory

## Abstract

While the implementation of the “graded diagnosis and treatment” system highlights the important role of general practitioners as “residents’ health gatekeepers”, it brings the problem of insufficient service capacity and difficulty in realizing the service value. At present, the service value of general practitioners is a relatively new topic in the field of general medicine. Therefore, few studies discuss the specific path that affects the realization of their service value. According to literature analysis, the professional quality of general practitioners plays a positive role in improving their service quality. So it can be inferred that the main reason for this phenomenon is that the professional quality level of general practitioners as the service subject is low and they have not been trusted and recognized by the residents of the service object. So far, it is difficult for most residents to change their willingness to go to large hospitals. Training is the most critical link to improving the professional quality of general practitioners. Therefore, how to enhance the professional quality of general practitioners through effective training so as to realize the service value is a problem worth discussing. Our study took 37 general practitioners from 12 Community Health Service hospitals as the interviewees and used grounded theory to mine the internal correlation between variables. The results show that: (1) the professional quality of general practitioners mainly includes three dimensions: professional ethics, theoretical knowledge, and professional skills; (2) through training, the professional quality of general practitioners has been effectively improved; (3) the improvement of general practitioners’ professional quality directly affects the realization of their technical value, environmental value and information value; (4) the professional quality of general practitioners can be improved through training, which will affect the realization of their service value. Our research contribution is to break through the previous research paradigm of analyzing the relationship between variables based on the existing literature. This paper uses the procedural grounded theory method to analyze the concept of general practitioners’ professional quality from scratch through continuous refinement and summary and constructs a theoretical model of the training path from general practitioners’ professional quality to service value. On the one hand, the research results can realize their service value by improving the professional quality of general practitioners. On the other hand, the realization of the service value of general practitioners can provide effective support for patients to create a good medical environment.

## 1. Introduction

As the “residents’ health gatekeepers”, general practitioners act as the first filter of the whole social–medical security system. Driven by this goal, the realization of the service value of general practitioners undertakes the front-end filtering function of the whole social–medical security system and is affected by the interaction of the serviceability of the back-end medical system. Therefore, the level of general practitioners’ serviceability in this situation determines whether they can shoulder this important task. At present, the implementation of policies such as “graded diagnosis and treatment” and “serious illness in hospital, minor illness in community” is difficult to promote, which reflects that the medical work of the general practitioner who is the “gatekeeper” of residents’ health has not been recognized and paid attention to by the residents. Therefore, there is a strong contrast between the reality and the expected service value. The main reason for this result is the low professional quality of general practitioners, which affects the promotion of service value and the implementation of policies. Specifically, the professional ethics, theoretical knowledge reserve, and professional skills of general practitioners are relatively low. Because the nature of the work of general practitioners is to switch back and forth between multiple identities, the professional ethics of doctors, such as being indifferent to fame and fortune, inner peace, and treating patients with a sincere attitude and good communication, are affected. Busy work will not allow general practitioners too much time to accumulate basic professional knowledge and other relevant knowledge. The development of professional skills, such as door-to-door service, is relatively slow. These phenomena reflect that the professional quality of general practitioners is at a relatively low level, which limits the realization of their service value, resulting in some difficulties in the implementation of the policy of “serious illness into the hospital and minor illness into the community”. Based on practical problems, the Chinese government has proposed a series of policies to improve the professional quality of general practitioners in community hospitals. In 2017, the National Development and Reform Commission’s “Thirteenth Five-Year Plan” National Health and Family Planning Professional Technical Personnel Training Program proposed the use of training resources in various ways to increase continuing medical education and training; in 2018, in the “opinions on reforming and improving the incentive mechanism for the training and use of general practitioners”, the Chinese government called for a reasonable allocation and expansion of the enrollment quota of standardized training for professional residents. It can be seen from the policy that training is not only aimed at continuing education for in-service general practitioners, but also has increased the amount of standardized training. Some scholars have also pointed out that gaining the trust of residents improves the basic knowledge and clinical competence of grassroots doctors [1,2,3]. Most of the literature indicates that training can improve the professional quality of general practitioners. Training programs are of great help to general practitioners’ consultation skills (reception, medical history collection, and medical record records); at the same time, they can increase the accumulation of theoretical knowledge [4]. More and more general practitioners find that relevant professional ethics should be added to the training, which is convenient for establishing effective communication with patients [5]. It can be seen that training is an important way to improve the professional quality of general practitioners, but the effectiveness test of the training results, that is, what service value it will bring, is not clear at present.

Professional quality refers to workers’ understanding and knowledge of occupation through social practice activities [6], and finally forms the qualities they need in the work. Generally, professional quality refers to comprehensive qualities such as knowledge, professional skills, and professional ethics. According to China’s current national conditions, the State Council of China puts forward the definition of general practitioners in the “Guiding Opinions” as: general practitioners are medical professionals with a high degree of comprehensiveness. They are mainly responsible for prevention and health care, diagnosis and treatment of common and frequently occurring diseases, referral, patient rehabilitation, chronic disease management, health management, and other integrated services at the grassroots level and are known as “residents’ health gatekeepers”. Through the understanding of professional quality and the definition of general practitioners, this research believes that the professional quality of general practitioners is the comprehensive quality of professional skills and professional ethics formed by general practitioners through their understanding of the integrated services of prevention and health care and health management [7,8,9,10].

Professional quality is generally used in education fields such as teachers and students. In most fields, competence is used as the standard to measure ability and quality. Competence is relatively widely used in enterprises, and scholars mainly divide its dimensions from the perspectives of knowledge, skills, communication, and professional ethics [11,12]. In the field of general medicine, most studies use general practitioners’ competence as the main measurement form. They mainly include the competency model of primary health care, community disease management, the ability to solve specific problems, and overall analysis [13,14]. At present, there are not many descriptions of the professional quality of general practitioners. Some scholars analyze the dimensions of professional quality from the perspective of clinical pharmacists, which mainly include professional ethics, professional skills, and professional behavior [15]. According to the available literature, how to improve the professional quality of general practitioners through training is the main concern of scholars [16,17,18]. At present, general practitioner training mainly includes three forms: standardized training, transfer training, and continuing education. The target population of standardized training is medical students, and the training focuses on basic theories, clinical skills, and primary medical and health practices [19,20]. In recent years, some scholars have proposed that humanistic quality should be added to the training, mainly starting from the physical and mental health of patients and paying attention to the correlation between humanistic emotion and management ability and the factors of patients’ physiological attributes [21,22,23]. Some trainees reported that the level of professional knowledge and skills, teamwork, and learning ability has improved, and community health service capabilities have been improved [24,25]. The transfer training is aimed at qualified basic-level on-the-job vocational physicians or vocational assistant physicians. The content of the transfer training is also carried out around the professional quality of general practitioners. It mainly includes training in knowledge and skills, and the emphasis on mental health and communication skills has also increased [26,27]. After training, appropriate adjustments should be made according to the service effect. For example, in the face of the application of general medicine and the unsatisfactory mastery of knowledge and skills in the field of preventive medicine. The main reason for this phenomenon is that the types of transfer trainees are complex, so it is necessary to carry out targeted training for trainees at different levels [28,29]. Continuing education is mainly for on-the-job general practitioners. The training contents mainly include clinical medical knowledge and skills, rational drug use, and disease diagnosis and treatment. The training has played a positive role in improving the comprehensive professional level [30,31,32]. The three forms of training face different groups of people, reflecting the breadth and comprehensiveness of the training. The content of the training is mainly carried out around the professional quality of general practitioners. After the training, it produces certain effects, such as knowledge and skills. All skills have improved, and service capabilities have also been improved [33,34,35]. Service value is reflected by the value perception of the demander and the value obtained by the supplier by satisfying the demander. There are many factors that affect the value of services, among which the quality of service and the service perception atmosphere on the demand side are two important aspects. For example, Sadeh et al. found through empirical research that the level of customer perceived service value is related to the quality of service provided by the enterprise [36]. Mokhtaran et al. assessed the impact of employees’ perceived service atmosphere and customers’ perceived service value [37]. Through the research on the service value measurement dimension, it is found that scholars gradually extend the technical value, functional value, cognitive value, and other dimensions from the perceived value of customers in the enterprise. In the field of general medicine, there is relatively little research on service value. For example, Wang constructed a community health service value evaluation model, which is divided into environmental value, information value, and technical value [38].

According to the above analysis, we can find that scholars have achieved some results in the research on the dimensional division of general practitioners’ professional quality and the relationship between professional quality and training, but there is a lack of relevant research on training to service value. At present, the research on service value dimensions and influencing factors is relatively rich, which can provide corresponding clues for the research on the relationship between training and service value. It can be seen from its influencing factors that although general practitioner training rarely directly affects service value, the above-mentioned service quality and service perception atmosphere of the demander are related to general practitioner training. The purpose of training is basically to improve the service quality of general practitioners so as to create a good service perception atmosphere for patients and ultimately realize their service value. Based on the above findings, there are few studies on the service value of general practitioners, and it is difficult to infer the specific process of its realization from the existing literature. Therefore, our study breaks through the research paradigm of deducing the relationship between professional quality, training, and service value based on the existing literature. The grounded theory method is to take the experience and thoughts described by the interviewees as the original data. Through the level-by-level coding of data, a higher-level category is extracted. It is a process of mining and discovering the internal relationship between the three from complex phenomena so as to build a theoretical model more in line with the actual development needs. Firstly, this paper introduces the grounded theory research method, research design, and theoretical sampling. Secondly, according to the research paradigm of procedural grounded theory, we conducted open coding, axial coding, and selective coding, and finally obtained the theoretical model of the training path from the professional quality of general practitioners to their service value by continuously extracting higher-level categories. Finally, the theoretical saturation test is carried out, and the coding results are analyzed in depth.

## 2. Methods

### 2.1. Grounded Theory

Grounded theory was jointly proposed by American sociologists Glaser and Strauss in the book the “Discovery of Grounded Theory: Strategies for Qualitative Research” [39]. The grounded theory mainly includes the classical grounded theory, the procedural grounded theory, and the Constructivist’s Approach to Grounded Theory. We chose the procedural grounded theory. Firstly, the advantage of grounded theory is to mine new theories from reality rather than verifying existing theories in the literature. Among them, the characteristic of procedural grounded theory is a way to establish theory from bottom to top, that is, to find the aggregation points from scattered data to form the core category and then connect the core categories into clues through data analysis and comparison, so as to finally form the overall model [40]. Compared with other research methods, although the subjectivity of the grounded theory method is slightly obvious, the preliminary research of hypothesis theory is very valuable. The procedural grounded theory research method emphasizes that the reasoning of the theoretical model presents a process of clear logic and complete structure. The specific steps are open coding, axial coding, and selective coding. These three levels of coding do not exist in isolation but complement each other. The strength of their internal logical association is an increasing trend. The previous level of coding is to lay the foundation for the next level of coding. At the same time, the coder needs to collect and compare data at different stages until it reaches theoretical saturation.

### 2.2. Research Design

This study uses the grounded theory method to construct a theoretical model of the training path from professional quality to service value of general practitioners. Raw data is mainly collected in the form of semi-structured interviews. The focus of this research is: (1) Refining the dimensions of general practitioners’ professional ethics. (2) Discuss the relationship between professional quality and training of general practitioners. (3) The service value mechanism of the professional quality of general practitioners through training. The initial interview outline consists of 6 questions: (1) What are your job content and responsibilities? (2) What professional qualities do you think are required to complete the above-mentioned work content? (3) In your work experience, what occupational confusions exist? Can you share 1–2 impressive career development experiences with us? (It can be successful, or it can be confused or low-pitched) (4) Which trainings have you participated in? What are the main methods and contents of these trainings? (5) After completing the training, how do you feel about the training process and effects? (You can express your personal feelings by sharing 1–2 training experiences) (6) Since you took office, what do you think the residents in the area have about general medical care? What is the patient’s perception of the unit’s general medical service? If there is a change, is it related to general practitioner training? Why? In the interview, according to the interviewee’s statement and the in-depth analysis of different levels of the problem, no other new problems appeared, indicating that the analysis of the problem has basically reached a relatively saturated state.

In order to realize the specific mechanism of the training path model from the professional quality of general practitioners to service value, the interview questions raised in this paper correspond to the conclusions. Firstly, how to obtain the professional quality of general practitioners? Secondly, what professional qualities are included in the training? Finally, what aspects of professional quality have been improved after the training, so as to realize what service values? Questions 1, 2, and 3 aim to obtain the conceptual model of the professional quality of general practitioners; Question 4 is about the content and form of training; Questions 5 and 6 aim to reflect that the professional quality of general practitioners has been improved to varying degrees after training, thus affecting the realization of service value.

### 2.3. Theoretical Sampling

These interviews were mainly in a face-to-face format between June to November 2021. The communication time between the interviewer and each interviewee was generally controlled within one hour, and the interview content was recorded through voice and notes with their consent. The interviewees are mainly represented by 8 community hospitals in Zhenjiang City, Jiangsu Province, including: Qilidian Community Health Service Center, Jingkou District Health Road Community Health Service Center, Jiangsu University Hospital, Huashan Bay Community Health Service Station, Baota Road Community Health Service Center, Runzhou District Sanmaogong Community Health Service Station, Baota Road Community Health Service Center, Liming Community Health Service center. In addition, it also includes 4 health service centers in other regions: Dongfeng Town Health Center, Liaoheyuan Town Central Hospital in Jilin Province, Shanghai Xuhui Longhua Street Community Health Service Center, Suzhou Gusu District Shuangta Street Zhonglou community health service station. There are 37 general practitioners in 12 hospitals. Take 24 bits from it (numbered 1–6, 8, 10–13, 16, 18, 22–24, 26, 28, 31–36) are used for model construction, and the remaining 13 bits (numbered 7, 9, 14, 15, 17, 19–21, 25, 27, 29, 30, 37) are used for model checking. The coding team set up in this paper includes two doctors and two masters. First, the interview recording is converted into editable text as the original data of coding. In order to reflect the interviewer’s real thoughts and states, we did not change the expression of the original sentence. Secondly, according to the research paradigm of procedural grounded theory, the original data are encoded in three levels—see below for details. During the whole coding process, we invited two senior theorists to answer all kinds of questions encountered in the coding process to ensure the validity and scientificity of the coding results.

## 3. Results

### 3.1. Open Coding

The purpose of this research is to build a training path model from professional quality to service value for general practitioners. In order to highlight the research question, it is necessary to delete the contents of the interviewee’s answer that are not related to this research. The initial open coding was carried out by integrating relevant data information. In the coding process, we randomly selected part of 37 respondents as the original data. Part of the data needs to be retained for the final theoretical model saturation test; therefore, we used 24 of the 37 general practitioners for modeling and the remaining 13 for theoretical model testing. In order to reflect the scientificity of coding, this study used Nvivo 10 qualitative analysis software to conduct a preliminary coding analysis of the text. During the first data processing, the statements related to this article are called free nodes. In total, 163 free nodes were obtained after excluding sentences irrelevant to this study. Through manual coding, we put forward keywords in 163 free points, classified and refined them in the way of clustering, and finally formed 18 clusters. In order to express the category of each cluster more clearly, we extracted a higher-level concept from each cluster. As shown in Table 1, after comparing and analyzing the contents of open coding, 18 frequent categories are obtained.

Among them, (1) “doctors themselves” in the main category of Table 1 mainly refers to the fact that in the face of lower salaries and fewer promotion opportunities, the quality of general practitioners indifferent to fame and wealth determines whether they can contribute to their work; doctor–patient conflict is inevitable. Keeping an inner peace attitude is not only an important way to alleviate this problem but is also a test of the inner tolerance of general practitioners; when faced with difficulties, clear goals and firm beliefs are an important manifestation of the excellent personal qualities of doctors. (2) The most fundamental aspect of “treating patients” in this study is to have a true and sincere attitude; communication is an important way for general practitioners to understand the health of patients and accurately analyze problems; most patients in community health hospitals are elderly people—the patience and encouragement of doctors are the driving force for patients to recover; when patients are in a disease state, emotions will inevitably fluctuate. As a general practitioner, you need to be sympathetic to the patient’s pain and understand empathy.

### 3.2. Axial Coding

Axial coding is based on open coding. It integrates scattered data and analyzes related content through cluster analysis to form different categories and establish connections, making the resulting code more complete and relevant and conceptualization, through detailed comparative and integrated analysis, the category formed by open coding (also known as the initial code)—“outpatient” and “door-to-door service” as the two main concept categories of “professional skill quality” in general practitioners’ professional quality subcategories; take the initial category—“book” and “network” as two subcategories of the main concept category of “theoretical knowledge” in the professional quality of general practitioners; take the initial category—“treating patients” and “doctors themselves” as two sub-categories of the main concept category of “professional ethics” in the professional quality of general practitioners; the initial categories—“regulation training”, “continuing education” and “transfer training” are regarded as the “training form” in general practitioners training three sub-categories of the main concept category; take the initial category—“national prescriptive training” and “community hospital autonomous training” as the two sub-categories of the main concept category of “training assessment” in general practitioners training; take the initial category—“moral quality”, “theoretical knowledge” and “practical skills” as the three subcategories of the main concept category of “training content” in general practitioner training; take the initial category—“humane environment atmosphere” as one sub-category of the main concept category of “environmental value” in the service value of general practitioners; the initial category—“skill level” and “patient satisfaction” are regarded as the main concept category of “technical value” in the service value of general practitioners two sub-categories; the initial category—“residential medical awareness” is regarded as a sub-category of the main concept category of “information value” in the service value of general practitioners. After repeated comparison and integration, nine conceptual categories (similar to secondary categories) were refined, as shown in Table 2.

After clustering and analyzing the data, it is found that the nine concepts obtained are related. According to the causal relationship and logical sequence, they are classified and summarized into three major types of relationships, as shown in Table 3.

### 3.3. Selective Coding

**Figure 1:**
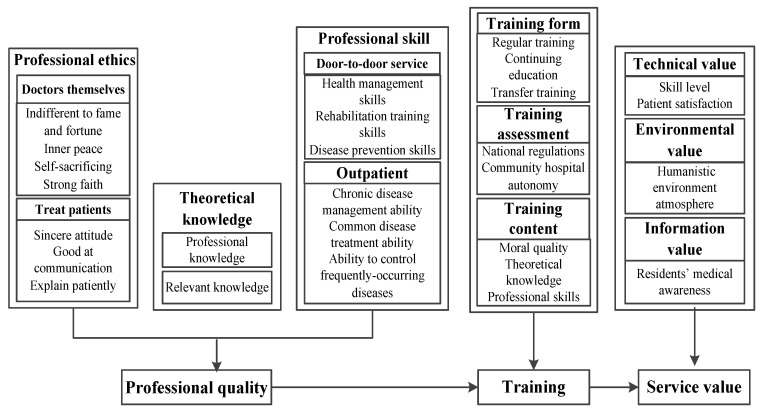
The training path model of general practitioners’ professional quality to its service value.

The purpose of this research is to construct the professional quality dimension of general practitioners as a basis to explore the influence pathways of professional quality on service value. Research on the training influences pathways of quality and service value. The core issue of the research can be conceptualized as a “research on the relationship between general practitioners’ professional quality, training, and service value ”. Figure 1 shows the core concept’s control structure over other concepts.

As shown in Figure 1, the internal relations between the concepts embodied in the “training path model of professional quality and service value of general practitioners” are as follows: (1) There is an inevitable connection between the concepts, which is mainly attributed to the discussion of professional ethics and theoretical knowledge in professional quality and the service value generated by the training of professional skills; (2) the three levels of professional quality: Internal morality–external knowledge theory–practical skills operation constitute the dimension of professional quality; (3) training is based on professional quality three dimensions are the main content. In terms of moral training, humanities such as inner peace, self-sacrificing, and good communication are mentioned. In terms of theoretical knowledge, special attention is paid to the training of new medical knowledge. Skills training is more prominent for new skills and first aid training; (4) after training, the service value of technology, environment, and information has been realized.

### 3.4. Theoretical Saturation Test

The researcher will use the data of 13 interviewees to test the theoretical saturation according to the three-level coding process. In this process, we have not found any new concept categories, and there is no relationship different from the established relationship between categories; therefore, it can be judged that the theoretical model is saturated. From more interview data, the researcher chooses the following six pieces of information as evidence:

(1) We will regularly visit the homes of rural residents, mainly to do some basic health management tasks, such as helping the elderly to regularly measure blood sugar and blood pressure, etc. For postoperative patients, we will also be responsible for rehabilitation training and guidance; I also observe the living conditions of the elderly and do a good job in disease prevention. (28-1-2 “door-to-door service”).
(2) Develop long-term management plans for patients with chronic diseases such as diabetes, hypertension, and heart disease. For frequently occurring patients with neck, shoulder, back, and leg pain, endocrine disorders, and disease control. For common colds, fevers, and gastritis, the sick patient has the ability to deal with the disease in time. (25-1-1 “outpatient”)
(3) Our regular prescriptions for chronic diseases are prescribed on a monthly basis, and he will come to you to prescribe them before he finishes his medicine. In this situation, we also try to explain patiently to patients. (9-3-2 “patience explain”)
(4) Our own unit will also organize some training, and there will be more training in general business. (9-4-1 “community hospital autonomous training”)
(5) After the training, you can indeed learn knowledge, gain some new knowledge, and use some new drugs. After all, you have graduated many years ago, and your knowledge is updated quickly. If you do not study, you will not be able to master it. (15-5-1 “theoretical knowledge”)
(6) After training, there will be a certain impact on the patient’s satisfaction. Because the patient is selective, he thinks that he will trust you after he has asked you a few times to solve the problem. I will come to you, may leave a phone call, and call you when I have something to do. (15-6-1 “patient satisfaction”)

## 4. Analysis of Coding Results

In this study, 37 general practitioners were interviewed in depth from 12 community health service hospitals, and the interview voice was transformed into the text as the original data. Then, the three-level coding forms (open coding, axial coding, and selective coding) followed the procedural grounded theory to refine higher-level categories step by step and connected the internal logical relationship between the professional quality, training, and service value of general practitioners. Finally, the “training path model of general practitioners’ professional quality and service value” is constructed.

(1) It has formed a three-dimensional dimension of the professional quality of general practitioners, that is, the quality of professional ethics, theoretical knowledge, and professional skills.
  First of all, noble professional ethics is the premise of being a qualified general practitioner and the soul of this profession. As a code of conduct, professional ethics are formed in the long-term medical practice of medical staff. It is the moral concept and moral behavior that every medical staff should have. Especially for general practitioners with strong service, it highlights the importance of their professional ethics. The biggest difference between general practitioners and specialists is that specialists manage diseases, while general practitioners manage people, and people-centered is the core concept of their practice. General practitioners mainly take the family as the unit, take the maintenance and promotion of overall health as the direction, and participate in the long-term physical and psychological life cycle responsible care process of individuals from birth to death. In this process, they play a variety of roles, such as educators, consultants, and health guardians. They are medical talents with a high degree of integration. It can be seen that it is extremely important to maintain long-term and effective communication with patients and always understand their needs, which puts forward higher requirements for the internal cultivation and noble professional ethics of general practitioners [41]. From the personal perspective of general practitioners, the reality of lower income and fewer promotion opportunities require them to have the noble character of weak fame and wealth, maintain inner peace for the complex nature of work, and have firm faith in the face of difficulties. Only general practitioners with these noble characteristics can contribute to their work. The formation of high professional ethics quality of general practitioners is a process of continuous accumulation and perception. It is a very key psychological dimension of professional quality. When general practitioners work and live in a high-intensity and stressful environment for a long time, they are prone to form negative emotions, such as anxiety and depression, and their mental health status will change to varying degrees. Especially in the recent outbreak of the new coronavirus epidemic, the general practitioner’s psychological endurance capacity has put forward higher requirements. It requires not only the general practitioner’s psychological state of self-sacrifice and firm belief, but also the need to consider the patient’s mood, communicate effectively and understand the importance of empathy.
  Secondly, in addition to mastering basic professional knowledge, such as internal medicine, surgery, gynecology, and pediatrics, general practitioners should also understand and learn other theoretical knowledge, such as psychology and interpersonal science, because the main feature of general practice is to serve patients with an overall medical view and a systematic way of thinking. For example, general practitioners should not only pay attention to the patient’s disease, but also observe the changes in the patient’s mood and the factors that may lead to the disease, such as the surrounding environment; they should even predict the possible diseases in the future according to the current patient’s physical condition—all of this to prevent the occurrence of diseases, prevent or shorten the time of future diseases in advance, or transfer patients in time according to the patient’s physical condition, and share the pressure of medical treatment for large hospitals [42,43]. This requires general practitioners to have a comprehensive reserve of theoretical knowledge in order to improve the accuracy of judging diseases and reduce the risk of miscalculation. In the process of growing up, general practitioners should also pay attention to their in-depth research on a certain aspect of professional knowledge. In this way, while training general practitioners to have the overall concept, they will also establish unique thinking required for hard research.
  Finally, the professional skills of general practitioners are not only the core component of professional quality, but also the embodiment of the practical workability of general practitioners. The professional skill quality of general practitioners mainly includes door-to-door service and outpatient service. In recent years, the state has gradually attached importance to door-to-door service, mainly because the aging population has led to the gradual increase of disabled, elderly people in China. In order to meet the medical needs of these patients, higher requirements are put forward for general practitioners’ health management skills, rehabilitation training skills, and disease prevention skills [44]. At present, there are many elderly people in community clinics. Chronic diseases (such as diabetes, hypertension, and heart disease) are very common diseases in the elderly. Therefore, general practitioners should have the ability to manage chronic diseases and control the development of patients’ diseases as much as possible. The other group is the residents near the community. Generally, people with common diseases (such as colds, headaches, etc.) will choose the community hospital close to home for treatment, which requires the general practitioner to have the ability to deal with common diseases. In addition, some patients suffer from frequently occurring diseases all year round (such as neck, shoulder, waist, and leg pain, endocrine disorders, etc.), and will also choose a more convenient community hospital to regulate their bodies. Therefore, general practitioners should have the ability to control the frequently occurring diseases of patients.
(2) The internal relationship model among general practitioners’ professional quality, training, and service value is established.
  Specifically, professional ethics, theoretical knowledge, and professional skills, as the dimensions of general practitioners’ professional quality, can be improved and improved through training, so as to further realize their technical value, environmental value, and information value. At present, the most important way to improve doctors’ professional quality and skills is to improve doctors’ theoretical and practical skills through training; therefore, the state should pay attention to vigorously carrying out the standardized training of general practitioners and strengthening the construction of grassroots health personnel.
  Training mainly includes standardized training, job transfer training, and continuing education. The standardized training is aimed at medical students. The training focuses on basic theories, clinical skills, and basic medical and health practice. Through training, most of the trainees reported that their professional knowledge level and skills, teamwork, and learning ability had been significantly improved, further promoting the realization of the technical value of general practitioners. The job transfer training is aimed at qualified grassroots on-the-job professional doctors or professional assistant doctors. The content of job transfer training is also carried out around the professional quality of general practitioners, mainly including knowledge, skills, and other training. The emphasis on mental health and communication skills has also increased. After the training, appropriate adjustments should be made according to the service effect. The training is gradually strengthening the application of general practice medicine and the content of knowledge and skills in the field of preventive medicine. As the types of trainees for job transfer training are complex, it is necessary to implement targeted training for trainees at different levels. Therefore, trained general practitioners are conducive to realizing their environmental value, technical value, and functional value. The improvement of general practitioners’ medical knowledge, medical experience, and ability to quickly handle emergencies is conducive to the further realization of technical value, and the improvement of family planning technical guidance, disease prevention, health care, and health management services is conducive to the further realization of functional value.
  The cultivation of humanistic quality needs long-term accumulation and precipitation. For general practitioners, the cultivation of humanistic quality is gradually concerned in training. Through the establishment of an assessment mechanism suitable for the innovative teaching model, more subjective training content is added, and students’ noble humanistic feelings, such as fraternity and empathy, are improved. In order to guide students to apply theory to practice, use the form of case analysis to simulate and restore the authenticity of real events, so as to constantly adjust and deepen the humanistic concept, which plays an important role in realizing good environmental values for general practitioners.

## 5. Conclusions

According to the research steps of the program-rooted theory, we constantly extract higher-level categories through the form of three-level coding, and finally refine the internal logical relationship between the professional quality, training, and service value of general practitioners, so as to build a training path model between the professional quality and service value of general practitioners.

Firstly, the concept of professional quality of general practitioners is deeply analyzed from the internal spiritual level of professional ethics to the material level of theoretical knowledge and then to the practical level of professional skills.

Secondly, training has an obvious impact on the professional quality of general practitioners. Training forms mainly include regular training, continuing education, and job transfer training. The assessment is organized by both national and community hospitals. The main content of the training is based on the professional ethics, theoretical knowledge, and professional skills of general practitioners. The coding results show that these three aspects of professional quality have been effectively improved through training.

Finally, it clarifies the internal relevance of the professional quality, training, and service value of general practitioners. The professional quality of general practitioners has been effectively improved through training, thus realizing their technical value, environmental value, and information value. The realization of technical value is due to the improvement of skill level, which increases patient satisfaction. The realization of environmental value is the promotion of moral quality, which shapes a good humanistic environment. The realization of information value is the acceptance and learning of new knowledge and advanced technology in the training process, which plays a positive role in improving residents’ medical cognition.

## 6. Theoretical Contribution and Practical Value

### 6.1. Theoretical Contribution

This paper breaks through the previous research paradigm of analyzing the relationship between variables based on the existing literature. We mainly use the procedural grounded theory method to mine and discover the internal relations between variables from complex phenomena based on real materials. Through continuous refinement and summary, a theoretical model more in line with the actual development needs can be established. Therefore, this article realizes the exploratory construction of the theoretical model of the training path from nothing to the service value of general practitioners. The theoretical model constructed in this study not only enriches the research in this field, but also provides a certain theoretical reference value for the research in other related fields.

### 6.2. Practical Value

The construction of the concept model of general practitioners’ professional quality not only provides a certain reference value for the government and community hospitals to make scientific management decisions and plans, but also provides directional guidance for general practitioners to clarify their own career growth. From the practical level, as the “residents’ health gatekeepers”, the professional quality of general practitioners is the basis for the implementation of the hierarchical diagnosis and treatment system. However, after the professional quality of general practitioners has been improved through training, the realization of their service value can be affected by increasing patients’ trust and influencing their medical behavior. This has certain practical significance to further implement the policy of “graded diagnosis and treatment”, and alleviate the problem of it being “expensive and difficult to see a doctor”.

## 7. Managerial Implications

Firstly, we scientifically arrange and optimize the training content and form so as to highlight the characteristics of general practice and meet the work and development needs of general practitioners so as to achieve the effect of learning for application and avoid the disconnection between the training content and actual work [45,46,47].

Secondly, in order to broaden the scope of theoretical knowledge learning, we make them master medical preface information by providing more opportunities to go to large hospitals, high-end universities, or study abroad, and bring the latest ideas to the community and patients, so as to promote the realization of their own information value; establish courses to improve communication skills, solve the language and other communication barriers between patients and doctors, actively learn foreign teaching experience, introduce standardized patients and adopt the form of scenario simulation to enhance the training effect, which is conducive to creating a good humanistic environment and realizing the environmental value of general practitioners.

Finally, in terms of training methods, case discussion, skill operation guidance, general practice teaching ward rounds, independent practice opportunities, and overall rotation department arrangement are mostly used to improve their professional ability. It is suggested that case teaching should be used in clinical rotation training to combine theory with practice. Training clinical thinking ability of general practitioners can fundamentally improve their clinical skills. This is conducive to the realization of its information value and technical value.

## 8. Limitations and Future Research

This study uses the grounded theory method to construct the “training path model of general practitioners’ professional quality and service value”, but there are still deficiencies. (1) The grounded theory method used in this study focuses on finding the internal relationship between variables in the way of logical deduction. This research paradigm has been considered as important by many scholars. At the same time, quantitative methods should be further used to measure the model in order to test its scientific nature. However, due to space constraints, this study did not verify this. (2) Although the grounded theory has no specific requirements for the number of data sources and only needs to reach the theoretical saturation, there may be a phenomenon that the research results will change with the increasing number of areas and people investigated. In future research, we will use the empirical method to test the scientificity and feasibility of the “training path model of general practitioners’ professional quality and service value”, and use the QCA method to explore pathways affecting the improvement of general practitioners’ professional quality, so as to better realize the service value from the perspective of improving the professional quality of general practitioners. This will be the main issue that we need to further study in the future.

## Figures and Tables

**Table 1:** Open coding category.

No	Main Category	Concept
1	Door-to-door service	Generally speaking, the interviewees said: Door-to-door service should have three aspects of skills and qualities: First, health management skills, including basic medical service capabilities such as blood pressure measurement, blood sugar measurement, and dressing change. The second is the rehabilitation training skills, the functional guidance to help disabled elderly, and the management of the condition of long-term bedridden patients. The third is disease prevention skills. Through long-term observation of the patient’s physical state and emotional changes, once an abnormal situation is found, the ability to guide the patient to accurately seek medical treatment in a timely manner.
2	Outpatient clinic	Disease types include: First, the ability to manage chronic diseases such as diabetes, hypertension, and heart disease. The second is the ability to control frequently occurring diseases such as neck, shoulder, waist, and leg pain and endocrine disorders. The third is the ability to deal with common diseases such as colds, fever, and gastritis.
3	Books	Generally, interviewees will choose to read books on basic medicine, such as general practitioner manuals, general medicine introductions, and also read books on psychology and doctor–patient communication to enrich their theoretical knowledge.
4	Internet	Download online resources such as Good Doctors.com and Dingxiang.com through the mobile APP to learn theoretical knowledge and broaden the ways of acquiring knowledge.
5	Treatment patients	The moral qualities that doctors need to treat patients: sincere attitude (2) *, good at communication (8), patient explanation (11), and empathy (3).
6	Doctors themselves	The doctor’s moral cultivation: indifferent to fame and fortune (1), inner peace (4), self-sacrifice (1), firm belief (2).
7	Regulation training	The regulation training time is three years, and content includes internal, external, women, children, health care, etc. During the study period, each department rotates to cultivate operational skills.
8	Continuous education	Participate in continuing education in the form of lectures and conferences every year.
9	Transfer of training	Learn theoretical knowledge in the form of class; you can choose a few basic courses, and rotate in the selected department about once every three months.
10	National mandatory training assessment	It is stipulated that the required credits can be completed before the assessment can be completed.
11	Autonomous training and assessment of community hospitals	Community hospitals regularly organize general practitioners to study or participate in lectures in major hospitals, and experts from major hospitals also regularly attend classes in community hospitals, forming an interactive training model.
12	Moral quality	General interviewees said: The professional ethics during the training period mainly involves communication skills, attitudes, people-oriented, belief-building, and other ideological content.
13	Theoretical knowledge	During the training, the professional knowledge learning mainly focuses on basic medical theory learning and the explanation of the latest development of chronic diseases in the community (such as diabetes, hypertension, etc.).
14	Practical skills	Skills and operation training mainly focuses on the mastery of basic operation skills and new technologies. The center is about lung rejuvenation, cardiovascular and cerebrovascular, and other first-aid operations.
15	Humanistic environment	The personal impact of the training on the general practitioner: The training is a summary explanation, with a deep memory point. In the work, I will often remind myself of the attitude and tone of communication with the patient to reduce misunderstandings and contradictions.
16	Skill levels	The personal impact of training on general practitioners: First, the knowledge level has enriched theoretical literacy and improved understanding of diseases. The second is the skill level, with a good understanding of new skills, especially in emergency training such as cardiopulmonary resuscitation. The improvement of doctors’ personal operating ability is beneficial to accurately determine the patient’s disease and provide effective treatment.
17	Patient satisfaction	Training effect: Increasing patients’ trust and recognition of general practitioners. Most interviewees said that they have fixed “fans” and come to see doctors regularly. As the quality of general practitioners in community hospitals has been improved through training, the number of outpatient clinics has shown an increasing trend year by year.
18	Residents’ medical awareness	Training effect: General practitioners have improved their own quality through training, mastered new knowledge and skills, and increased their awareness of general medical care in the process of communicating with patients.

Note: * indicates that the number in parentheses refers to the frequency of the vocabulary and similar vocabulary in all sorted out sentences of interviewees.

**Table 2:** Main concept categories and content.

No	Main Category	Concept
1	Professional ethics quality	It summarizes the professional ethics quality of general practitioners from two aspects: treating patients and doctors themselves.
2	Theoretical knowledge	Theoretical knowledge is a necessary professional quality for general practitioners, and books and the Internet are important ways for them to obtain basic content.
3	Professional skills quality	The form of outpatient service requires general practitioners to have the ability to manage chronic diseases, control frequently occurring diseases, and handle common diseases. The form of door-to-door service requires general practitioners to have health management skills, rehabilitation training skills, and disease prevention skills.
4	Training form	The training forms mainly include regular training, continuing education, and transfer training.
5	Evaluate training	The assessment units are national training assessment and community hospital independent training assessment
6	Training content	The training content is related to the professional quality of general practitioners, including three aspects: ethics, theoretical knowledge, and skills.
7	Technical value	The training enriches the theoretical knowledge of general practitioners and effectively improves their practical skills, especially the improvement of operating skills for handling emergencies. At the same time, the increase in patient satisfaction has prompted a continuous increase in the number of outpatient services.
8	Environmental value	Through the humanistic qualities formed by the exchanges and communication between doctors and patients after training, a good environmental atmosphere is created for community residents.
9	Information value	In the training process, general practitioners accept advanced knowledge and can provide general medical service information and medical progress in time, so that they can effectively answer questions raised by residents.

**Table 3:** Three types of relationships based on axial coding.

No	Relationship Category	Concept of Influence Relationship (Corresponding Code)	Relationship Connotation
1	Professional quality	Professional ethics quality: Doctors themselves ① indifferent to fame and fortune (1-3-1) ② inner peace (1-3-2, 10-3-2, 4-3-2, 24-3-2) ③ self-sacrificing (1-3-4) ④ strong faith (24-3-1, 22-3-1); Treat patients ① sincere attitude (3-3-1, 6-3-2) ② good at communication (1-3-4, 2-3-2, 6-3-3, 11-3-1, 16-3-1, 18-3-2, 22-3-2, 33-3-2) ③ explain patiently (1-3-3, 5-3-3, 10-3-1, 12-3-4, 13-3-3, 23-3-1, 24-3-3, 28-3-2, 32-3-1, 35-3-5, 36-3-2) ④ empathy (2-3-1, 3-3-2, 28-3-1)	The most fundamental thing for a doctor is to have a heart of “the benevolent loves others”, and a good professional ethics is the inner soul of the spiritual level of a doctor. From the doctor’s point of view, indifference to fame and fortune, inner peace, self-sacrifice, and firm conviction are the basic characteristics of personal charm. A sincere attitude, good communication, patient explanation, and empathy are the good moral qualities that general practitioners need to have when facing patients. In addition to internal literacy, general practitioners also need to have profound theoretical knowledge, professional skills, and other external qualities.
		Theoretical knowledge: books (5-4-2, 20-4-2, 26-4-4, 35-4-4); network (8-4-3, 26-4-5, 4-4-3, 35-4-2, 31-4-3)	
		Professional skill quality: Outpatient ① chronic disease management ability (1-1-1, 8-1-1, 10-1-1, 12-1-1, 13-1-1, 22-1-1, 23-1-1, 24-1-1, 26-1-1, 28-1-1, 33-1-1, 34-1-1, 35-1-1) ② common disease treatment ability (2-1-1, 3-1-1, 5-1-1, 6-1-1, 11-1-1, 16-1-1, 18-1-1, 24-1-1, 26-1-1, 31-1-1, 32-1-1, 35-1-1, 36-1-1) ③ ability to control frequently occurring diseases (4-1-1); Door-to-door service ① health management skills (2-1-2, 5-1-2, 12-1-4, 23-1-2, 28-1-2, 31-1-2, 32-1-2, 36-1-2) ② rehabilitation training skills (1-1-3, 4-1-2, 11-1-2, 28-1-2) ③ disease prevention skills (28-1-2)	
2	Training	Training form: regular training (8-4-3, 10-4-1, 12-4-1, 36-4-2); continuing education (6-4-2, 12-4-4, 16-4-2, 24-4-1, 32-4-1, 33-4-2); transfer training (13-4-1, 18-4-1, 33-4-1)	The professional quality of general practitioners determines the trust and satisfaction of patients. Training is an effective way to improve one’s own professional quality, mainly involving three aspects of training form, assessment, and content. The main forms of training are planned training, continuing education, and transfer training. The assessment is organized by both the state and community hospitals. The training content is mainly set according to the basic quality of general practitioners.
		Training assessment: National regulations (1-4-4, 4-4-1, 5-4-1, 8-4-1); community hospital autonomy (1-4-5, 4-4-2, 5-4-3, 10-4-2, 11-4-2, 16-4-4, 22-4-2, 34-4-2)	
		Training content: moral quality (1-4-2, 2-4-2, 5-4-4, 26-4-3, 28-4-2, 31-4-1, 35-4-5, 36-4-3); theoretical knowledge (13-4-5, 23-4-2, 26-4-2, 32-4-2, 34-4-3); skills (1-4-3, 2-4-1, 8-4-2, 10-4-3, 11-4-1, 26-4-1, 28-4-1, 35-4-3)	
3	Service value	Technical value: skill level (11-5-3, 13-5-3, 16-5-1, 26-5-1, 35-5-1, 34-5-2, 32-5-1, 31- 5-1, 2-5-1, 3-5-1, 6-5-1, 10-5-1, 11-5-1, 24-5-1, 34-5-1, 31-5-2, 6-5-2); patient satisfaction (11-6-4, 12-6-2, 24-6-3, 26-6-3, 2-6-1, 3-6-1, 22 -6-1, 28-6-2, 31-6-3, 36-6-2)	Through training, the technical value, environmental value, and information value of general practitioners have been realized. The realization of technical value is due to the improvement of skill level, which increases patient satisfaction. The realization of environmental value is the promotion of moral quality, which has shaped a good humanistic environment. The realization of the value of information is the acceptance and learning of new knowledge and advanced technology in the training process, which has a certain positive effect on the improvement of residents’ medical awareness.
		Environmental value: humanistic environment atmosphere (2-5-2, 11-5-2, 35-5-3, 36-5-1)	
		Information value: residents’ medical awareness (1-6-1, 10-6-2, 13-6-1, 33-6-1)	

## Data Availability

The raw data supporting the conclusion of this article will be made available by the authors upon reasonable request.
